# Trophic Position and Metabolic Rate Predict the Long-Term Decay Process of Radioactive Cesium in Fish: A Meta-Analysis

**DOI:** 10.1371/journal.pone.0029295

**Published:** 2012-01-18

**Authors:** Hideyuki Doi, Teruhiko Takahara, Kazuya Tanaka

**Affiliations:** Institute for Sustainable Sciences and Development, Hiroshima University, Higashi-Hiroshima, Japan; University of British Columbia, Canada

## Abstract

Understanding the long-term behavior of radionuclides in organisms is important for estimating possible associated risks to human beings and ecosystems. As radioactive cesium (^137^Cs) can be accumulated in organisms and has a long physical half-life, it is very important to understand its long-term decay in organisms; however, the underlying mechanisms determining the decay process are little known. We performed a meta-analysis to collect published data on the long-term ^137^Cs decay process in fish species to estimate biological (metabolic rate) and ecological (trophic position, habitat, and diet type) influences on this process. From the linear mixed models, we found that 1) trophic position could predict the day of maximum ^137^Cs activity concentration in fish; and 2) the metabolic rate of the fish species and environmental water temperature could predict ecological half-lives and decay rates for fish species. These findings revealed that ecological and biological traits are important to predict the long-term decay process of ^137^Cs activity concentration in fish.

## Introduction

Atmospheric nuclear tests in the 1960 s emitted large amounts of radionuclides, including radioactive strontium (^90^Sr), iodine (^131^I), and cesium (^134^Cs, ^137^Cs). In particular, the uptake of such radionuclides into human bodies is of serious concern [1]–[3]. Therefore, the long-term behavior of radionuclides in the environment is an important issue for estimating possible radiological consequences and associated risks [4]. Because cesium and potassium have similar chemical natures to alkali elements, radioactive cesium can be also accumulated in organisms, especially in muscle tissues [5]–[7].

**Figure 1:**
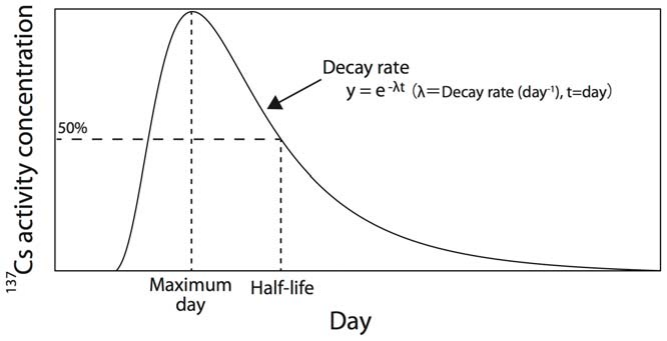
Illustration of the conceptual decay processes of ^137^Cs activity concentration in fish bodies.

Because the decay of ^137^Cs emits γ-rays (662 keV), its accumulation inside bodies causes internal radiation exposure [8]. Cesium-137 (physical half-life: 30.07 years) is a long-lived radionuclide relative to ^131^I and ^134^Cs (8.02 days and 2.07 years, respectively). It is important to know how long accumulated ^137^Cs stays inside the body and when it is excreted from the body (i.e., biological decay of ^137^Cs) [5], [7]–[9]. The decay process of radioactive metals (e.g., ^137^Cs) in the body has been well understood (Fig. 1); however, the underlying mechanisms shaping the decay are little known. Potentially, two major drivers can shape the decay: biological and ecological traits of organisms.

Biological traits influencing the decay process of radioactive metals include, for example, the uptake and excretion rates of ^137^Cs. The metabolic theory of ecology (MTE), which considers the whole metabolic process of organisms, has been recently developed [10]–[12]. MTE can predict the whole metabolic rate of an organism from its body mass and temperature [11]. For aquatic species, excluding mammals, the body temperature is determined by the absolute temperature of the environment inhabited by the species. The metabolic rate (B, unit: Watt) can be predicted by B = b_0_M^0.75^e^−E/kT^ [11], where b_0_ is the normalization constant independent of body size and temperature, and M, E, k, and T are body mass (g), the activation energy, Boltzmann's constant, and absolute temperature in degrees kelvin (K), respectively. Metabolic rate would affect the decay process, as some studies have indicated that body mass is an important factor determining long-term trends in decay [11]. However, these studies did not fully consider MTE with body mass and temperature to predict decay trends.

Ecological factors including the trophic and habitat traits of species have been considered as drivers of the accumulation of ^137^Cs in the body. Trophic relationships have been identified as important drivers of material bioaccumulation in various ecosystems because trophic transfer through food webs accumulates material concentrations in the consumer body [1], [13], [14]. Thus, species with higher trophic position would have increased activity concentration of radioactive cesium [15]. A meta-analysis paper tested this hypothesis for concentration factor (CF; i.e. ^137^Cs_species_/^137^Cs_backgound_) and suggested that trophic transfer accumulates ^137^Cs and increases the CF of higher-trophic species [16]. Habitat effects on the accumulation and decay of ^137^Cs have not been well tested, but a few studies have shown differences in CF between species inhabiting pelagic and bottom aquatic habitats. Many studies have reported the radionuclide CF of organisms [16]–[19]. However, the effects of trophic and habitat traits on the long-term decay processes of radioactive metals have not been well tested [15].

From the above knowledge, we can hypothesize that ecological and biological traits such as trophic position, habitat type, and metabolic rate would predict the decay process of ^137^Cs in organisms. This hypothesis has been partly tested on single species or groups of species within an ecosystem and under laboratory conditions, but the generality of the hypothesis across species has never been tested, despite the importance of general patterns in ecological and biological effects on the decay process for predicting the decay process of unstudied species as well as whole communities.

In this study, we focused on fish taxa to test the above hypothesis because many studies related to ^137^Cs activity concentration in fish bodies have been published, especially after the Chernobyl fallout accident of 1986, and the Fishbase dataset can provide the data on trophic position and the other ecological traits (habitat and diet types) for estimating the effects of ecological traits on the decay process. From a fisheries viewpoint, the ^137^Cs activity concentration of fish is significant to human health if humans consume fish that have been collected from radiation-polluted areas of marine and freshwater ecosystems [3].

Here, we performed a meta-analysis using published datasets to estimate how ecological and biological traits determine the long-term decay processes of ^137^Cs in fish bodies. From this analysis, we can reveal the general underlying mechanisms of the long-term decay processes and predict the decay processes of radioactive cesium in fish bodies.

## Materials and Methods

### Data collection

We searched data from published sources using the ISI Web of Science and Google Scholar. The search term was “(accum* or concent*) AND (radioacti* or cesium or strontium) AND fish”. The search was conducted on 24 April 2011, and returned 291 and 1,071 studies from Web of Science and Google Scholar, respectively. Also, we checked the journal and gray references in the searched papers. Most studies found using the key word searches described natural cesium (^133^Cs) and strontium (Sr) concentrations in fish. From the searched references, we selected the papers containing long-term data on the changes in radioactive cesium or strontium in fish bodies in natural ecosystems and laboratory experiments; we collected data from the texts, figures, and tables of the papers. To gather data from figures, we used PlotDigitizer X ver. 2.0.1 software (available: http://www.surf.nuqe.nagoya-u.ac.jp/#x7e;nakahara/Software/PlotDigitizerX/). In total, we obtained 260 long-term data items from 34 papers (total 58 fish species, Table S1). Some species were represented by multiple data points (see Table S1). We searched the data on radioactive strontium (^90^Sr) concentrations in fish, but obtained only two long-term data items; thus, we analyzed only the data on radioactive cesium.

We extracted 3 indices of long-term decay trends in ^137^Cs activity concentrations in fish bodies (Fig. 1). Maximum day (unit: day): the day when the maximum ^137^Cs activity concentration was recorded in the fish body after the input of ^137^Cs into the ecosystem or experiment. Ecological half-life (hereafter half-life, unit: day): the day when the activity concentration had decreased to 50% of its maximum activity concentration in the fish body. The physical half-life of ^137^Cs is 30.07 year, but the ecological half-lives were relatively shorter (646.8±828.1 days, mean±1 SD); thus, the effects of chemical disintegration would be ignorable. Decay rate (day^−1^): the coefficient of exponential regression during decreasing ^137^Cs activity concentration. We found other long-term indices such as uptake rate; however, the sample sizes were very small, and thus we used the above three indices for the analyses.

Ecological and biological data (trophic position, diet types, habitat types, and average body mass) were obtained from the original papers, from the Fishbase database (http://www.fishbase.org), or from other sources. The mean water temperature of the water body during monitoring or year was obtained from the original paper (almost all data are annual mean temperature). If the original paper did not have the temperature data, we got the annual mean temperature of the water body from World Lake Database (http://wldb.ilec.or.jp/) or other sources.

### Body mass and temperature

Fish body mass and ambient water temperatures from the datasets were used to estimate the metabolic rates of species. From the model of metabolic theory (see Introduction, [11]), we tested the metabolic-rate effect on the decay process of ^137^Cs activity concentrations in fish bodies using their body mass and water temperature. In this study, water temperature was used as inverse temperature, 1/kT, where k and T are Boltzmann's constant (8.62×10^−5^ eV K^−1^) and absolute water temperature in degrees Kelvin (K).

### Statistical analyses

Generalized linear mixed models (GLMMs, [20]) were performed to predict the 3 indices of long-term trends, including maximum day, half-life and decay rate (α = 0.05). We included the possible explanatory factors for the GLMMs: trophic position, habitat types (as category), diet types (as category), body mass, and inverse temperature (1/kT). We used the Gaussian distribution as the error distribution for the GLMM, and species and the data collection (laboratory or field investigations) were treated as the random factors to consider the variations among the species and the data collection methods. The maximum day, half-life, decay rate, and body mass were log_10_ transformed to normalize the values. We selected the best GLMMs by a downward stepwise procedure based on the Akaike Information Criterion (AIC) [21], [22]. We also calculated AIC differences (Δ*i*) and Akaike weight (ω*_i_*), which is considered as the weight of evidence in favor of a candidate model being the best model out of the set of models considered [23]. Before the GLMM analysis, we calculated the variance inflation factor (VIF) to check for co-linearity of the factors. The maximum VIF was 1.73 for all models, indicating that co-linearity among the factors would not significantly influence the results of GLMMs. All data used for statistical analyses are provided in Table S1. All statistics and graphics were performed using R ver. 2.13.0 [24].

## Results

### Trophic position, body mass, and inverse temperature affect the three indices

**Figure 2:**
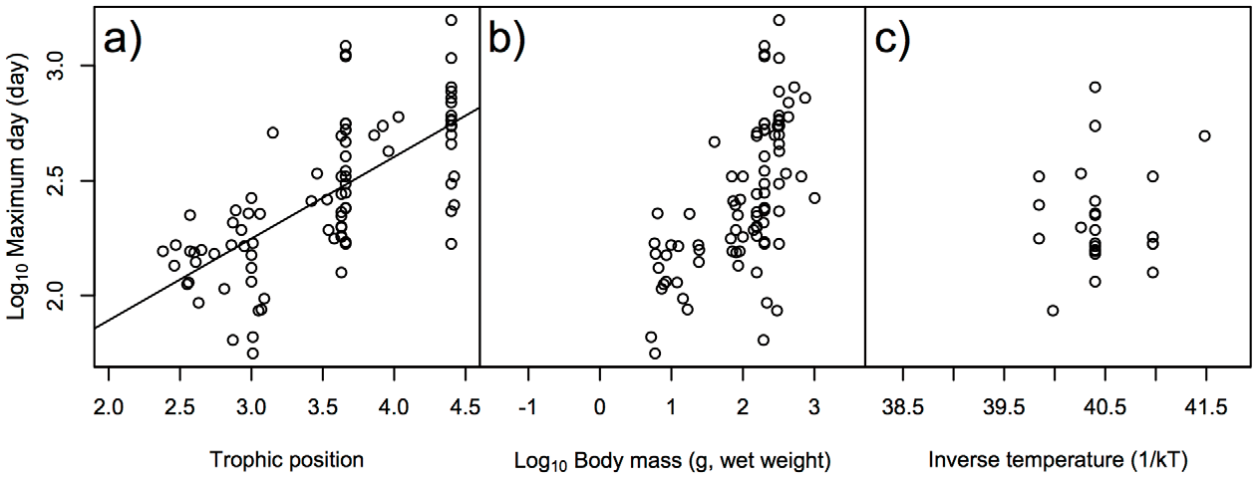
Effects of ecological/biological traits of species on maximum day of ^137^Cs activity concentration in fish. Effects of a) trophic position of species, b) body mass (g, wet weight) of species, and c) inverse temperature (1/kT) of the environment on log_10_ maximum day (day) of ^137^Cs activity concentration in fish. Line indicates a significant relationship by the best GLMM, with the coefficient and intercept estimated by GLMM.

**Figure 3:**
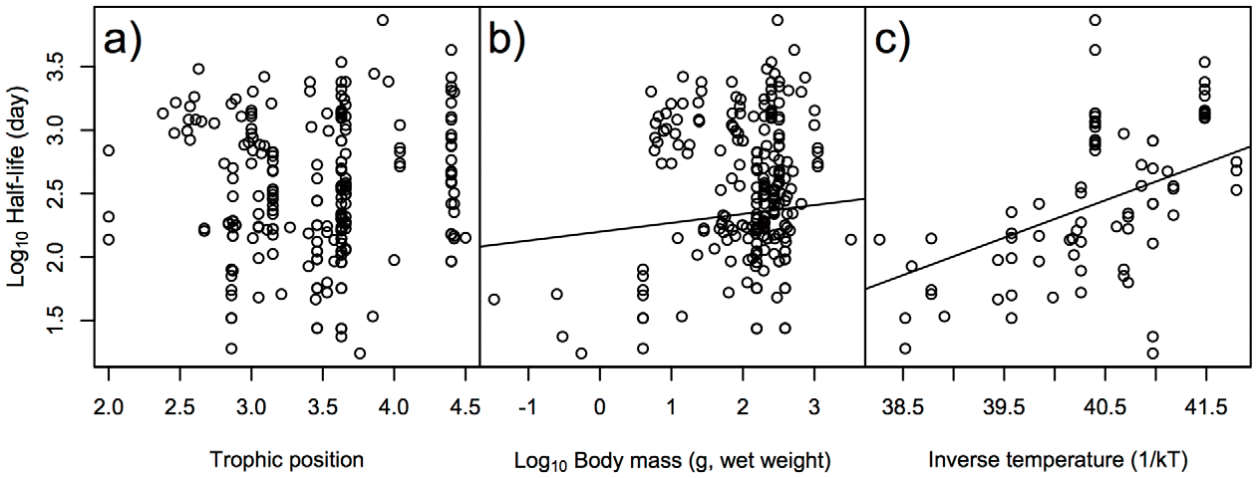
Effects of ecological/biological traits of species on half-lives of ^137^Cs activity concentration in fish. Effects of a) trophic position of species, b) body mass (g, wet weight) of species, and c) inverse temperature (1/kT) of the environment on log_10_ half-lives (day) of ^137^Cs activity concentration on fish. Lines indicate a significant relationship by the best GLMM, with the coefficient and intercept estimated by GLMM.

**Figure 4:**
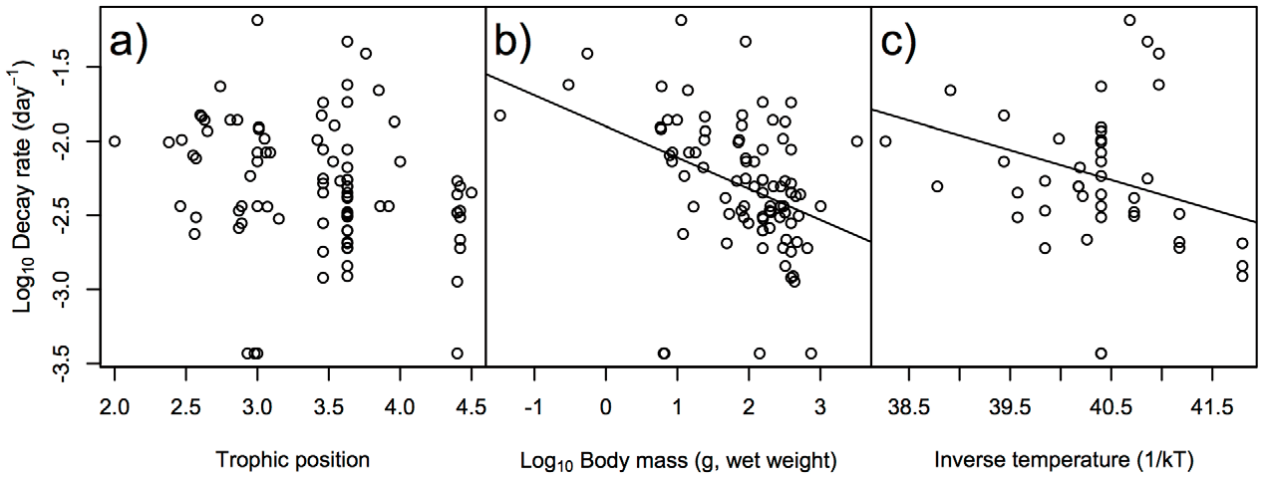
Effects of ecological/biological traits of species on decay rate of ^137^Cs activity concentration in fish. Effects of a) trophic position of species, b) body mass (g, wet weight) of species, and c) inverse temperature (1/kT) of the environment on log_10_ decay rate (day^−1^) on ^137^Cs activity concentration in fish. Lines indicate a significant relationship by the best GLMM, with the coefficient and intercept estimated by GLMM.

For the maximum day of ^137^Cs activity concentration in fish bodies, the best GLMM only included trophic position as the explanatory factor (Table 1), and the other factors were not retained in the best model. The models including body mass and inverse temperature had also lower AIC and higher Akaike weights (ω*_i_*) values like the best model (Table 1). The relationship between maximum day and trophic position was significantly positive (Fig. 2, GLMM, *p*<0.001). For half-life days, the best GLMM included both body mass and inverse temperature as explanatory factors (Table 2). The models including ecological traits such as habitat types had relatively lower AIC values, but the coefficients were very low (Table 2). The half-lives were significantly positively correlated with both body mass and inverse temperature (Fig. 3, GLMM, *p*<0.01), indicating that the half-life of ^137^Cs activity concentration was shorter in species with higher metabolic rates. For decay rate, the best GLMM included both body mass and inverse temperature as explanatory factors (Table 3). The models including diet types had relatively lower AIC values, but the coefficient was very low (Table 3). The decay rate showed significantly negative correlations with both body mass and inverse temperature (Fig. 4, GLMM, *p*<0.01), indicating that species with higher metabolic rates had higher decay rates of ^137^Cs activity concentration.

**Table 1:** Parameter coefficients for log_10_ maximum day of ^137^Cs activity concentration in fish bodies estimated by all stepwised models of GLMM.

	Maximum day (day)					
Factors	Full	Step1	Step2	Step3	Step4	Best
Trophic position	**0.16**	**0.15**	**0.18**	**0.15**	**0.19**	**0.36**
Diet type	−0.01					
Habitat type	−0.02	−0.003	0.01			
Log_10_ Body mass (g)	0.05	0.05		0.05	0.05	
Inverse Temperature (1/kT)	0.06	0.05	0.05	0.05		
(Intercept)	**−0.72**	**−0.38**	**−0.29**	**−0.31**	**−0.45**	**1.18**
AIC	3.51	1.65	0.11	−0.10	−1.88	−2.17
Δ*i*	5.68	3.82	2.28	2.07	0.29	0.00
ω*_i_*	0.02	0.05	0.12	0.13	0.32	0.36

The models were selected by a downward stepwise procedure using Akaike Information Criteria (AIC). AIC, AIC differences (Δ*i*), and Akaike weights (ω*i*) were shown. Coefficients in bold indicate significant values at the 0.05 level.

**Table 2:** Parameter coefficients for log_10_ half-life of ^137^Cs activity concentration in fish bodies estimated by all stepwised models of GLMM.

	Half lives (day)			
Factors	Full	Step1	Step2	Best
Trophic position	−0.05	−0.05		
Diet type	0.02			
Habitat type	−0.05	−0.05	−0.07	
Log_10_ Body mass (g)	**0.14**	**0.15**	**0.14**	**0.14**
Inverse Temperature (1/kT)	**0.28**	**0.28**	**0.28**	**0.26**
(Intercept)	**−8.97**	**−9.03**	**−9.12**	**−8.48**
AIC	88.06	86.60	84.88	83.41
Δ*i*	4.65	3.19	1.47	0.00
ω*_i_*	0.03	0.06	0.15	0.32

The models were selected by a downward stepwise procedure using Akaike Information Criteria (AIC). AIC, AIC differences (Δ*i*), and Akaike weights (ω*i*) were shown. Coefficients in bold indicate significant values at the 0.05 level.

**Table 3:** Parameter coefficients for log_10_ decay rate (day^−1^) of ^137^Cs activity concentration in fish bodies estimated by all stepwised models of GLMM.

	Decay rate (day^−1^)			
Factors	Full	Step1	Step2	Best
Trophic position	−0.04	−0.04		
Diet type	−0.08	−0.08	−0.05	
Habitat type	0.00			
Log_10_ Body mass (g)	**−0.25**	**−0.25**	**−0.28**	**−0.28**
Inverse Temperature (1/kT)	**−0.28**	**−0.28**	**−0.27**	**−0.27**
(Intercept)	**10.00**	**10.00**	**8.99**	**9.03**
AIC	46.38	45.05	44.38	43.13
Δ*i*	3.25	1.92	1.25	0.00
ω*_i_*	0.09	0.18	0.25	0.47

The models were selected by a downward stepwise procedure using Akaike Information Criteria (AIC). AIC, AIC differences (Δ*i*), and Akaike weights (ω*i*) were shown. Coefficients in bold indicate significant values at the 0.05 level.

### Habitat and diet type effects on the three indices

**Figure 5:**
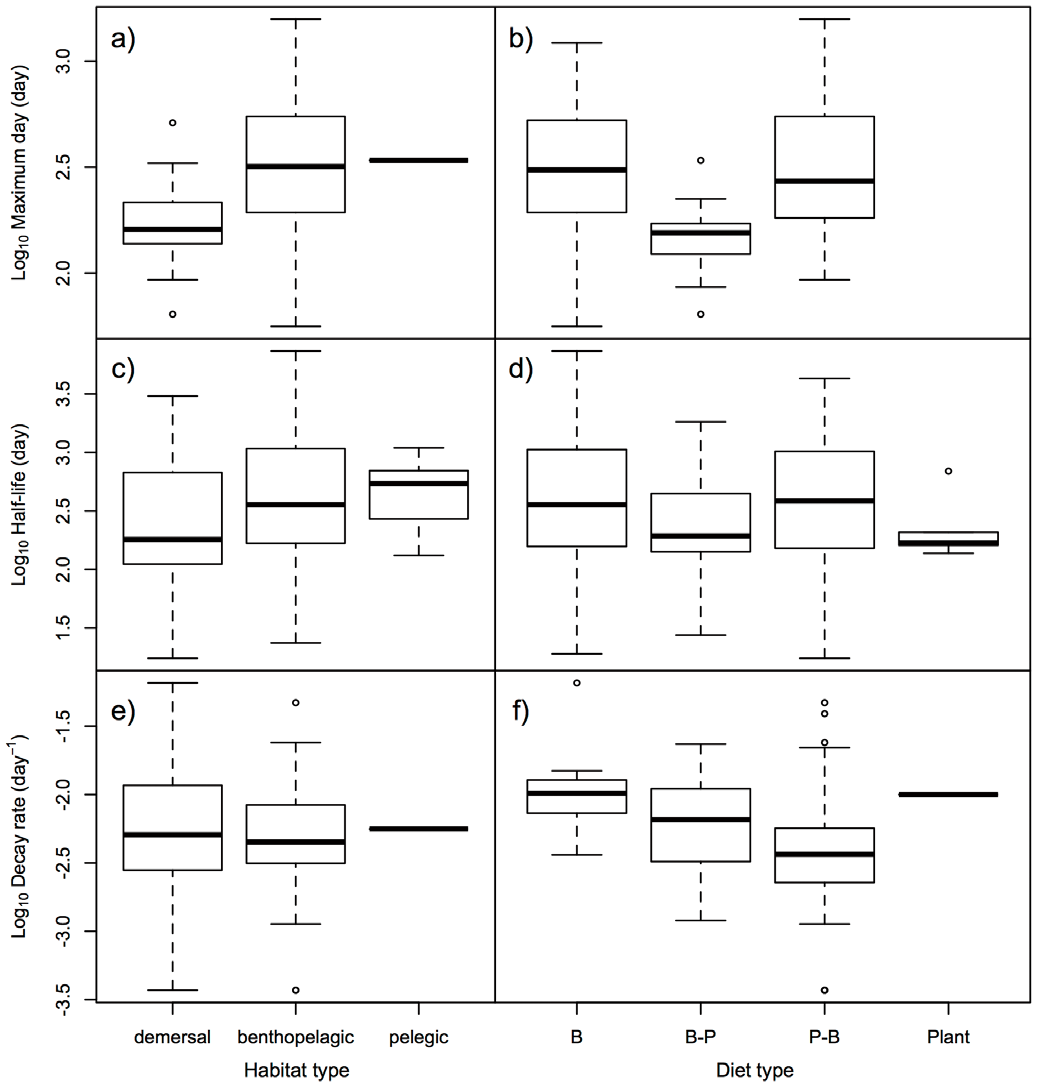
The long-term trends and habitat/diet types of species. Box and whisker plots of the indices of long-term trends (maximum day, half-lives, and decay rate) for different habitat and diet types of fish species. For diet types, B, B-P, P-B, and plant refer to benthos, benthos and plankton (benthos>plankton), plankton and benthos (plankton>benthos), and plant. Bars in the boxes, upper and lower box edges, and error bars indicate median, ±25% quantile, and 1.5×±25% quantile, respectively. There were no significant differences among the types for all indices (see Tables 1, 2, and 3).

As can be seen from the GLMMs of all indices (Tables 1, 2, and 3), habitat and diet types of the species did not significantly influence the indices and were not included as explanatory factors in the best GLMMs. The median values and the upper and lower quantiles of the indices overlapped remarkably (Fig. 5). Habitat and diet types did not significantly affect the decay process of ^137^Cs activity concentration in the bodies of fish species.

## Discussion

We found that the long-term decay process of ^137^Cs activity concentration in fish bodies was affected by trophic position and metabolic rate, which were determined by body mass and inverse temperature of the species. Such ecological/biological traits of species can predict the long-term decay process of ^137^Cs activity concentration in various fish species, as we hypothesized.

The maximum day of ^137^Cs activity concentration in the fish body was strongly correlated with trophic position. In freshwater systems, fish accumulate ^137^Cs mainly from their diets, and little from the water (<20%, [25]). Trophic transfer from primary producers to predatory fish would delay the maximum day of activity concentration in higher trophic species. After ^137^Cs was added into systems, the ^137^Cs activity concentration in primary producers increased, and then ^137^Cs was transferred to higher trophic species up the food chain. A time lag in the maximum day among species would influence the decay process of ^137^Cs activity concentration in the organisms in whole ecosystems because the ^137^Cs in dead fish would be decomposed by the microbial community and recycled in the ecosystems.

Traditionally, higher trophic-level species are frequently fishery species that are collected and consumed by humans [26]. Thus, after a radioactive pollution event, the radioactivity levels of higher trophic-level species should be carefully monitored [2], [18], [27]. In this study, we found that the coefficient of trophic position in the GLMM for log_10_ maximum day was 0.36; thus, the difference in maximum day between trophic levels 3 and 4 is about 230 days. Thus, when observing the monitoring data for lower trophic species, a time lag can be expected before the maximum ^137^Cs activity concentration of higher trophic-level fish species is reached.

Half-lives and decay rates were related to body size and inverse temperature, and positive/negative trends were observed. Thus, half-life and decay rate can be explained by the metabolic rate of the species. The metabolism of an organism includes the uptake of resources, the exclusion of resources, and the energy/growth ratio [11], [28]. Thus, the decay process that reduces ^137^Cs activity concentration is determined by uptake and exclusion rates. Also, body mass and inverse temperature had marginal positive effects on the maximum day of ^137^Cs activity concentration, probably because the metabolic rate of the species, such as the uptake and exclusion rates, also influenced to maximize ^137^Cs activity concentration in fish body. In this study, inverse temperature showed a clearer association with half-life and decay rate than did body mass. Thus, water temperature may be the better parameter for predicting half-lives and decay rates. Sundbom et al. [15] suggested that the half-lives of lake fish species are not related to trophic position but to body size. From our analyses, trophic position and other ecological traits were not found to significantly influence half-lives and decay rates. According to their suggestion, we also conclude that ecological traits including trophic relationships are not important for predicting the half-lives and decay rates of ^137^Cs activity concentration, but metabolic processes can predict half-lives and decay rates for many fish species.

The decay process of background ^137^Cs activity concentration in water and sediment would also influence the decay process in fish bodies [4], [29], [30]. Due to the dynamic equilibrium of ^137^Cs between inputs and outputs of the systems, the ecological half-life is not constant and varied over time within the same environment and species [31]. Because we gained few data on the background decay indices of ^137^Cs activity concentration, we could not estimate the importance of the background effect in predicting ^137^Cs activity concentration in fish. The decay process of ^137^Cs activity concentration in water is related to the dispersal rate and retention time of the water body. In this study, we estimated data from >83 water bodies with various volumes and locations; thus, the general patterns of decay process that we showed here would not be strongly influenced by local dispersal rate and retention time of water. However, further study is needed to consider the detailed interactions between the background and organism decay processes of ^137^Cs activity concentration. Although potassium concentration influences the ^137^Cs activity concentration of fish body [9], [16], we also cannot evaluate potassium effect on the decay processes of ^137^Cs, because we have the limited data for potassium concentration of the fish. Comparing marine and freshwater studies would evaluate the potassium concentration effect on ^137^Cs activity concentration in fish body, because of the different potassium concentrations in the marine and freshwater water. However, in our dataset, most data are from freshwater ecosystems, with very few data points from marine ecosystems. Thus, further study is needed to estimate the decay process of marine fish species, which are more important for fisheries, and to consider the dispersal of ^137^Cs to unpolluted regions due to marine fish migration.

In conclusion, our meta-analysis found that the trophic position and metabolic rates of fish species could predict the long-term decay process of radioactive cesium. As ^137^Cs activity concentrations in fish are potentially useful for assessing human risks from fish consumption, such predictions would be useful in policy making for the conservation of aquatic ecosystems after radiation fallout and for preserving human health by avoiding radiation exposure caused by eating fish.

## Supporting Information

Table S1 The dataset using the meta-analysis.
(DOC)
