# Supplementary material for: Trophic Position and Metabolic Rate Predict the Long-Term Decay Process of Radioactive Cesium in Fish: A Meta-Analysis
Source: PLoS One. 2012 Jan 18;7(1):e29295. doi: 10.1371/journal.pone.0029295 (PMC3261150; doi:10.1371/journal.pone.0029295)
Supplement: Table S1 — The dataset using the meta-analysis. (DOC) [file pone.0029295.s001.doc]

Table S1 The dataset using the meta-analysis. Paper number is reference number, which is listed below. Max day, Half-lives and Decay rate means the maximum day (day), half-live (day) and decay rate (day^-1^) of ^137^Cs in fish body. Mass, TP and WT mean average body mass (g, wet weight), trophic position and annual mean water temperature (°C).

| Paper | Species name | Species order | Max day | Half-lives | Decay rate | Mass | TP | Diet type | Habitat | Ecosystem | WT |
| --- | --- | --- | --- | --- | --- | --- | --- | --- | --- | --- | --- |
| 1 | *Micropogon undulatus* | Perciformes |  | 95 | 0.0073 | 119 | 4.00 | plankton-benthos | demersal | marine | 21 |
| 1 | *Paralichthys dentatus* | Pleuronectiformes |  | 46 | 0.0149 | 0.033 | 3.45 | benthos | demersal | marine | 21 |
| 2 | *Onchorhynchus mykiss* | Salmoniformes |  | 940 | 0.0655 | 11.3 | 3.00 | benthos | benthopelagic | freshwater | 12 |
| 3 | *Salmo trutta* | Salmoniformes |  | 3420 |  | 250 | 3.63 | plankton-benthos | demersal | freshwater | 6.5 |
| 3 | *Salmo trutta* | Salmoniformes |  | 2383 |  | 250 | 3.63 | plankton-benthos | demersal | freshwater | 6.5 |
| 3 | *Salmo trutta* | Salmoniformes |  | 2088 |  | 250 | 3.63 | plankton-benthos | demersal | freshwater | 6.5 |
| 3 | *Salmo trutta* | Salmoniformes |  | 1854 |  | 250 | 3.63 | plankton-benthos | demersal | freshwater | 6.5 |
| 3 | *Salmo trutta* | Salmoniformes |  | 1424 |  | 250 | 3.63 | plankton-benthos | demersal | freshwater | 6.5 |
| 3 | *Salmo trutta* | Salmoniformes |  | 1387 |  | 250 | 3.63 | plankton-benthos | demersal | freshwater | 6.5 |
| 3 | *Salmo trutta* | Salmoniformes |  | 1351 |  | 250 | 3.63 | plankton-benthos | demersal | freshwater | 6.5 |
| 3 | *Salmo trutta* | Salmoniformes |  | 1325 |  | 250 | 3.63 | plankton-benthos | demersal | freshwater | 6.5 |
| 3 | *Salmo trutta* | Salmoniformes |  | 1263 |  | 250 | 3.63 | plankton-benthos | demersal | freshwater | 6.5 |
| 3 | *Salmo trutta* | Salmoniformes |  | 1237 |  | 250 | 3.63 | plankton-benthos | demersal | freshwater | 6.5 |
| 3 | *Salmo trutta* | Salmoniformes | 496 |  |  | 156 | 3.63 | plankton-benthos | demersal | freshwater | 6.5 |
| 4 | *Esox* | Esociformes | 501 | 1203 |  | 320 | 4.40 | plankton-benthos | demersal | freshwater |  |
| 4 | *Esox* | Esociformes | 582 | 867 |  | 320 | 4.40 | plankton-benthos | demersal | freshwater |  |
| 4 | *Rutilus* | Cypriniformes | 208 | 417 |  | 194 | 2.87 | benthos-plankton | benthopelagic | freshwater |  |
| 4 | *Rutilus* | Cypriniformes | 64 | 301 |  | 194 | 2.87 | benthos-plankton | benthopelagic | freshwater |  |
| 4 | *Scardinius erythrophthalmus* | Cypriniformes |  | 179 |  | 98.5 | 2.89 | benthos-plankton | benthopelagic | freshwater |  |
| 4 | *Abramis brama* | Cypriniformes |  | 167 |  | 157 | 3.15 | plankton-benthos | benthopelagic | freshwater |  |
| 4 | *Esox* | Esociformes |  | 152 |  | 320 | 4.40 | plankton-benthos | demersal | freshwater |  |
| 4 | *Esox* | Esociformes |  | 93 |  | 320 | 4.40 | plankton-benthos | demersal | freshwater |  |
| 5 | *Esox lucius* | Esociformes |  |  |  | 475 | 4.40 | plankton-benthos | demersal | freshwater |  |
| 6 | *Salmo trutta* | Salmoniformes | 198 | 356 |  | 156 | 3.63 | plankton-benthos | demersal | freshwater | 15 |
| 6 | *Salvelinus alpinus* | Salmoniformes | 340 | 132 |  | 400 | 3.46 | benthos-plankton | pelagic | freshwater | 15 |
| 7 | *Perca fluviatilis* | Perciformes | 330 | 2390 |  | 70 | 3.66 | benthos | demersal | freshwater |  |
| 7 | *Salmo trutta* | Salmoniformes | 180 | 825 |  | 100 | 3.63 | plankton-benthos | demersal | freshwater | 10 |
| 7 | *Salmo trutta* | Salmoniformes | 330 | 825 |  | 100 | 3.63 | plankton-benthos | demersal | freshwater | 10 |
| 8 | *Salvelinus alpinus* | Salmoniformes |  | 1238 | 0.006 | 90 | 3.46 | benthos-plankton | pelagic | freshwater | 10.8 |
| 8 | *Salmo trutta* | Salmoniformes |  | 365 | 0.047 | 90 | 3.63 | plankton-benthos | demersal | freshwater | 10.8 |
| 9 | *Onchorhynchus mykiss* | Salmoniformes |  | 63 |  | 115 | 3.53 | benthos | benthopelagic | freshwater | 11.7 |
| 10 | *Salvelinus alpinus* | Salmoniformes |  | 278 |  | 389 | 3.46 | benthos-plankton | benthopelagic | freshwater |  |
| 10 | *Salmo trutta* | Salmoniformes |  | 200 |  | 156 | 3.63 | plankton-benthos | demersal | freshwater |  |
| 10 | *Salvelinus alpinus* | Salmoniformes |  | 179 |  | 389 | 3.46 | benthos-plankton | benthopelagic | freshwater |  |
| 10 | *Salvelinus alpinus* | Salmoniformes |  | 111 |  | 389 | 3.46 | benthos-plankton | benthopelagic | freshwater |  |
| 10 | *Salmo trutta* | Salmoniformes |  | 111 |  | 156 | 3.63 | plankton-benthos | demersal | freshwater |  |
| 10 | *Salvelinus alpinus* | Salmoniformes |  | 96 |  | 389 | 3.46 | benthos-plankton | benthopelagic | freshwater |  |
| 10 | *Salmo trutta* | Salmoniformes |  | 91 |  | 156 | 3.63 | plankton-benthos | demersal | freshwater |  |
| 10 | *Salmo trutta* | Salmoniformes |  | 91 |  | 156 | 3.63 | plankton-benthos | demersal | freshwater |  |
| 10 | *Salvelinus alpinus* | Salmoniformes |  | 57 |  | 389 | 3.46 | benthos-plankton | benthopelagic | freshwater |  |
| 10 | *Salmo trutta* | Salmoniformes |  | 57 |  | 156 | 3.63 | plankton-benthos | demersal | freshwater |  |
| 10 | *Salvelinus alpinus* | Salmoniformes |  | 27 |  | 389 | 3.46 | benthos-plankton | benthopelagic | freshwater |  |
| 10 | *Salmo trutta* | Salmoniformes |  | 27 |  | 156 | 3.63 | plankton-benthos | demersal | freshwater |  |
| 11 | *Sander lucioperca* | Perciformes |  | 1095 |  | 1000 | 4.04 | plankton-benthos | pelagic | freshwater |  |
| 11 | *Perca fluviatilis* | Perciformes |  | 548 |  | 10 | 3.00 | plankton-benthos | demersal | freshwater |  |
| 12 | *Perca fluviatilis* | Perciformes |  | 188 |  | 200 | 3.66 | benthos | demersal | freshwater | 15 |
| 12 | *Rutilus rutilus* (*Leuciscus rutilus*) | Perciformes |  | 78 |  | 195 | 2.87 | benthos-plankton | benthopelagic | freshwater | 15 |
| 12 | *Oncorhynchus mykiss* (*Salmo iridaeus*) | Salmoniformes |  | 53 |  | 63 | 3.53 | benthos | benthopelagic | freshwater | 15 |
| 13 | *Cyprinus carpio* | Cypriniformes |  | 174 |  | 137 | 3.05 | benthos-plankton | benthopelagic | freshwater | 12.5 |
| 13 | *Cyprinus carpio* | Cypriniformes |  | 98 |  | 137 | 3.05 | benthos-plankton | benthopelagic | freshwater | 20 |
| 14 | *Lepomis macrochirus* | Perciformes |  | 137 |  | 60 | 3.58 | benthos-plankton | benthopelagic | freshwater | 15.8 |
| 15 | *Hypophthalmichthys molitrix* | Cypriniformes |  | 138 | 0.01 | 3250 | 2.00 | plant | benthopelagic | freshwater | 30 |
| 16 | *Esox lucius* | Esociformes |  | 464 |  | 320 | 4.40 | plankton-benthos | demersal | freshwater |  |
| 16 | *Cyprinus carpio* | Cypriniformes |  | 303 |  | 415 | 3.05 | benthos-plankton | benthopelagic | freshwater |  |
| 16 | *Barbus perspensis* | Cypriniformes |  | 281 |  |  |  |  |  | freshwater |  |
| 16 | *Cyprinus carpio* | Cypriniformes |  | 219 |  | 415 | 3.05 | benthos-plankton | benthopelagic | freshwater |  |
| 16 | *Scardinius acarnanicus* | Cypriniformes |  | 208 |  | 57 | 2.00 | plant | benthopelagic | freshwater |  |
| 16 | *Rutilus rutilus doiranensis* | Cypriniformes |  | 193 |  | 195 | 2.87 | benthos-plankton | benthopelagic | freshwater |  |
| 16 | *Perka fluviatilis* | Perciformes |  | 190 |  | 200 | 3.66 | benthos | demersal | freshwater |  |
| 16 | *Rutilus rubilio* | Cypriniformes |  | 186 |  | 51 | 2.84 | benthos-plankton | benthopelagic | freshwater |  |
| 16 | *Perka fluviatilis* | Perciformes |  | 183 |  | 200 | 3.66 | benthos | demersal | freshwater |  |
| 16 | *Rutilus rubilio* | Cypriniformes |  | 183 |  | 51 | 2.84 | benthos-plankton | benthopelagic | freshwater |  |
| 16 | *Oncorhynchus mykiss* (*Salmo gairdneri*) | Salmoniformes |  | 175 |  | 63 | 3.53 | benthos | benthopelagic | freshwater |  |
| 16 | *Cyprinus carpio* | Cypriniformes |  | 172 |  | 415 | 3.05 | benthos-plankton | benthopelagic | freshwater |  |
| 16 | *Rutilus rutilus doiranensis* | Cypriniformes |  | 172 |  | 195 | 2.87 | benthos-plankton | benthopelagic | freshwater |  |
| 16 | *Tinca tinca* | Cypriniformes |  | 172 |  | 112 | 3.27 | benthos | demersal | freshwater |  |
| 16 | *Luciobarbus* [*Barbus*] *albanicus* | Cypriniformes |  | 168 |  | 28 | 2.67 | plant | benthopelagic | freshwater |  |
| 16 | *Carassius caracius gibelio* | Cypriniformes |  | 164 |  | 71 | 3.11 | plankton-benthos | demersal | freshwater |  |
| 16 | *Luciobarbus* [*Barbus*] *albanicus* | Cypriniformes |  | 161 |  | 28 | 2.67 | plant | benthopelagic | freshwater |  |
| 16 | *Oncorhynchus mykiss* (*Salmo gairdneri*) | Salmoniformes |  | 157 |  | 50 | 3.53 | benthos | benthopelagic | freshwater |  |
| 17 | *Perca fluviatilis* | Perciformes |  | 1095 |  | 70 | 3.66 | benthos | demersal | freshwater |  |
| 17 | *Blicca bjoerkna* | Cypriniformes |  | 755 |  | 214 | 3.09 | benthos | demersal | freshwater |  |
| 17 | *Sander lucioperca* | Perciformes |  | 724 |  | 1113 | 4.04 | plankton-benthos | pelagic | freshwater |  |
| 17 | *Hypophthalmichthys molitrix* | Cypriniformes |  | 692 |  | 48 | 2.00 | plant | benthopelagic | freshwater |  |
| 17 | *Sander lucioperca* | Perciformes |  | 674 |  | 1113 | 4.04 | plankton-benthos | pelagic | freshwater |  |
| 17 | *Abramis brama* | Cypriniformes |  | 673 |  | 157 | 3.15 | plankton-benthos | benthopelagic | freshwater |  |
| 17 | *Abramis brama* | Cypriniformes |  | 665 |  | 157 | 3.15 | plankton-benthos | benthopelagic | freshwater |  |
| 17 | *Abramis brama* | Cypriniformes |  | 664 |  | 157 | 3.15 | plankton-benthos | benthopelagic | freshwater |  |
| 17 | *Sander lucioperca* | Perciformes |  | 550 |  | 1113 | 4.04 | plankton-benthos | pelagic | freshwater |  |
| 17 | *Sander lucioperca* | Perciformes |  | 518 |  | 1113 | 4.04 | plankton-benthos | pelagic | freshwater |  |
| 17 | *Abramis brama* | Cypriniformes |  | 484 |  | 157 | 3.15 | plankton-benthos | benthopelagic | freshwater |  |
| 17 | *Perca fluviatilis* | Perciformes |  | 417 |  | 70 | 3.66 | benthos | demersal | marine |  |
| 17 | *Salvelinus alpinus* | Salmoniformes |  | 417 | 0.0012 | 389 | 3.46 | benthos-plankton | benthopelagic | freshwater |  |
| 17 | *Salvelinus alpinus* | Salmoniformes |  | 278 | 0.0018 | 389 | 3.46 | benthos-plankton | benthopelagic | freshwater |  |
| 17 | *Salmo trutta* | Salmoniformes |  | 263 | 0.0019 | 156 | 3.63 | plankton-benthos | demersal | freshwater |  |
| 17 | *Salmo trutta* | Salmoniformes |  | 200 | 0.0025 | 156 | 3.63 | plankton-benthos | demersal | freshwater |  |
| 17 | *Salmo trutta* | Salmoniformes |  | 200 | 0.0025 | 156 | 3.63 | plankton-benthos | demersal | freshwater |  |
| 17 | *Rutilus rutilus* | Cypriniformes |  | 192 | 0.0026 | 195 | 2.87 | benthos-plankton | benthopelagic | freshwater |  |
| 17 | *Salvelinus alpinus* | Salmoniformes |  | 179 | 0.0028 | 389 | 3.46 | benthos-plankton | benthopelagic | freshwater |  |
| 17 | *Scardinius erythrophthalmus* | Cypriniformes |  | 179 | 0.0028 | 99 | 2.89 | benthos-plankton | benthopelagic | freshwater | 14 |
| 17 | *Abramis brama* | Cypriniformes |  | 167 | 0.003 | 157 | 3.15 | plankton-benthos | benthopelagic | freshwater | 14 |
| 17 | *Salmo trutta* | Salmoniformes |  | 161 | 0.0031 | 156 | 3.63 | plankton-benthos | demersal | freshwater |  |
| 17 | *Esox lucius* | Esociformes |  | 152 | 0.0033 | 320 | 4.40 | plankton-benthos | demersal | freshwater | 14 |
| 17 | *Esox lucius* | Esociformes |  | 152 | 0.0033 | 320 | 4.40 | plankton-benthos | demersal | freshwater | 14 |
| 17 | *Rutilus rutilus* | Cypriniformes |  | 147 | 0.0034 | 195 | 2.87 | benthos-plankton | benthopelagic | freshwater | 14 |
| 17 | *Sprattus sprattus* | Clupeiformes |  | 141 |  | 12 | 3.01 | plankton-benthos | pelagic | marine |  |
| 17 | *Salmo trutta* | Salmoniformes |  | 111 | 0.0045 | 156 | 3.63 | plankton-benthos | demersal | freshwater |  |
| 17 | *Salvelinus alpinus* | Salmoniformes |  | 111 | 0.0045 | 389 | 3.46 | benthos-plankton | benthopelagic | freshwater |  |
| 17 | *Salvelinus alpinus* | Salmoniformes |  | 96 | 0.0052 | 389 | 3.46 | benthos-plankton | benthopelagic | freshwater |  |
| 17 | *Esox lucius* | Esociformes |  | 93 | 0.0054 | 320 | 4.40 | plankton-benthos | demersal | freshwater | 14 |
| 17 | *Salmo trutta* | Salmoniformes |  | 91 | 0.0055 | 156 | 3.63 | plankton-benthos | demersal | freshwater |  |
| 17 | *Salmo trutta* | Salmoniformes |  | 91 | 0.0055 | 156 | 3.63 | plankton-benthos | demersal | freshwater |  |
| 17 | *Salmo trutta* | Salmoniformes |  | 57 | 0.0088 | 156 | 3.63 | plankton-benthos | demersal | freshwater |  |
| 17 | *Salvelinus alpinus* | Salmoniformes |  | 57 | 0.0088 | 389 | 3.46 | benthos-plankton | benthopelagic | freshwater |  |
| 17 | *Salvelinus alpinus* | Salmoniformes |  | 27 | 0.0182 | 389 | 3.46 | benthos-plankton | benthopelagic | freshwater |  |
| 17 | *Salmo trutta* | Salmoniformes |  | 27 | 0.0183 | 156 | 3.63 | plankton-benthos | demersal | freshwater |  |
| 18 | *Silurus asotus* | Siluriformes |  | 142 | 0.0045 | 391 | 4.50 | plankton-benthos | ｄemersal | freshwater | 20 |
| 19 | *Salmo trutta* | Salmoniformes |  | 24 | 0.024 | 0 | 3.63 | plankton-benthos | demersal | freshwater | 10 |
| 19 | *Salmo salar* | Salmoniformes |  | 17 | 0.039 | 1 | 3.76 | plankton-benthos | benthopelagic | freshwater | 10 |
| 20 | *Lepomis gulosus* | Perciformes |  | 2383 |  | 26 | 3.41 | benthos | demersal | freshwater |  |
| 20 | *Notropis cummingsae* | Cypriniformes |  | 2201 |  |  |  |  | benthopelagic | freshwater |  |
| 20 | *Lepomis punctatus* | Perciformes |  | 2161 |  |  |  |  | demersal | freshwater |  |
| 20 | *Lepomis auritus* | Perciformes |  | 2022 |  | 26 | 3.41 | benthos | demersal | freshwater |  |
| 20 | *Micropterus salmoides* | Perciformes |  | 1993 |  | 657 | 4.42 | plankton-benthos | benthopelagic | freshwater |  |
| 20 | *Esox americanus* | Esociformes |  | 1745 |  | 93 | 3.65 | benthos-plankton | demersal | freshwater |  |
| 20 | *Aphredoderus sayanus* | Percopsiformes |  | 1617 |  | 14 | 3.14 | benthos | demersal | freshwater |  |
| 21 | *Salmo trutta* | Salmoniformes |  | 500 |  | 156 | 3.63 | plankton-benthos | demersal | freshwater | 10 |
| 22 | *Micropterus salmoides* | Perciformes |  | 320 | 0.0022 | 333 | 4.42 | plankton-benthos | benthopelagic | freshwater | 15 |
| 22 | *Micropterus salmoides* | Perciformes |  | 226 | 0.0031 | 271 | 4.42 | plankton-benthos | benthopelagic | freshwater | 20 |
| 22 | *Micropterus salmoides* | Perciformes |  | 140 | 0.005 | 279 | 4.42 | plankton-benthos | benthopelagic | freshwater | 26 |
| 23 | *Ictalurus punctatus* | Siluriformes |  | 154 |  | 151 | 3.40 | benthos-plankton | benthopelagic | freshwater | 20 |
| 23 | *Ictalurus punctatus* | Siluriformes |  | 85 |  | 151 | 3.40 | benthos-plankton | benthopelagic | freshwater | 27.5 |
| 24 | *Micropterus salmoides* | Perciformes | 330 | 263 | 0.0019 | 657 | 4.42 | plankton-benthos | benthopelagic | freshwater | 18 |
| 24 | *Micropterus salmoides* | Perciformes | 248 | 147 | 0.0034 | 78 | 4.42 | plankton-benthos | benthopelagic | freshwater | 18 |
| 24 | *Lepomis macrochirus* | Perciformes | 177 | 93 | 0.0054 | 68 | 3.58 | benthos-plankton | benthopelagic | freshwater | 18 |
| 24 | *Erimyzon sucetta* | Cypriniformes | 86 | 48 | 0.0104 | 300 | 3.05 | benthos-plankton | demersal | freshwater | 17 |
| 25 | *Esox* | Esociformes |  | 2194 |  | 320 | 4.40 | plankton-benthos | demersal | freshwater |  |
| 25 | *Perca* | Perciformes |  | 1278 |  | 200 | 3.66 | benthos | demersal | freshwater |  |
| 25 | *Esox* | Esociformes |  | 1267 |  | 320 | 4.40 | plankton-benthos | demersal | freshwater |  |
| 25 | *Anguilliformes* | Anguilliformes |  | 1051 |  |  |  |  |  | freshwater |  |
| 25 | *Perca* | Perciformes |  | 1007 |  | 200 | 3.66 | benthos | demersal | freshwater |  |
| 25 | *Salmo* | Salmoniformes |  | 1004 |  | 320 | 4.40 | plankton-benthos | demersal | freshwater |  |
| 25 | *Esox* | Esociformes |  | 920 |  | 320 | 4.40 | plankton-benthos | demersal | freshwater |  |
| 25 | *Esox* | Esociformes |  | 818 |  | 320 | 4.40 | plankton-benthos | demersal | freshwater |  |
| 25 | *Esox* | Esociformes |  | 748 |  | 320 | 4.40 | plankton-benthos | demersal | freshwater |  |
| 25 | *Perca* | Perciformes |  | 653 |  | 200 | 3.66 | benthos | demersal | freshwater |  |
| 25 | *Coregonus lavaretus* | Salmoniformes |  | 628 |  | 273 | 3.15 | benthos-plankton | demersal | freshwater |  |
| 25 | *Salmo* | Salmoniformes |  | 544 |  |  |  |  |  | freshwater |  |
| 25 | *Rutilus* | Cypriniformes |  | 504 |  | 194 | 2.87 | benthos-plankton | benthopelagic | freshwater |  |
| 25 | *Coregonus lavaretus* | Salmoniformes |  | 475 |  | 273 | 3.15 | benthos-plankton | demersal | freshwater |  |
| 25 | *Esox* | Esociformes |  | 445 |  | 320 | 4.40 | plankton-benthos | demersal | freshwater |  |
| 25 | *Esox* | Esociformes |  | 394 |  | 320 | 4.40 | plankton-benthos | demersal | freshwater |  |
| 25 | *Perca* | Perciformes |  | 369 |  | 200 | 3.66 | benthos | demersal | freshwater |  |
| 25 | *Coregonus lavaretus* | Salmoniformes |  | 343 |  | 273 | 3.15 | benthos-plankton | demersal | freshwater |  |
| 25 | *Perca* | Perciformes |  | 339 |  | 200 | 3.66 | benthos | demersal | freshwater |  |
| 25 | *Coregonus lavaretus* | Salmoniformes |  | 329 |  | 273 | 3.15 | benthos-plankton | demersal | freshwater |  |
| 25 | *Perca* | Perciformes |  | 314 |  | 200 | 3.66 | benthos | demersal | freshwater |  |
| 25 | *Salmoniformes* | Salmoniformes |  | 310 |  |  |  |  |  | freshwater |  |
| 25 | *Coregonus lavaretus* | Salmoniformes |  | 310 |  | 273 | 3.15 | benthos-plankton | demersal | freshwater |  |
| 25 | *Coregonus lavaretus* | Salmoniformes |  | 292 |  | 273 | 3.15 | benthos-plankton | demersal | freshwater |  |
| 25 | *Coregonus lavaretus* | Salmoniformes |  | 285 |  | 273 | 3.15 | benthos-plankton | demersal | freshwater |  |
| 25 | *Anguilliformes* | Anguilliformes |  | 281 |  |  |  |  |  | freshwater |  |
| 25 | *Coregonus lavaretus* | Salmoniformes |  | 256 |  | 273 | 3.15 | benthos-plankton | demersal | freshwater |  |
| 25 | *Coregonus lavaretus* | Salmoniformes |  | 234 |  | 273 | 3.15 | benthos-plankton | demersal | freshwater |  |
| 25 | *Coregonus lavaretus* | Salmoniformes |  | 219 |  | 273 | 3.15 | benthos-plankton | demersal | freshwater |  |
| 25 | *Salmoniformes* | Salmoniformes |  | 208 |  |  |  |  |  | freshwater |  |
| 25 | *Perca* | Perciformes |  | 168 |  | 200 | 3.66 | benthos | demersal | freshwater |  |
| 25 | *Coregonus lavaretus* | Salmoniformes |  | 150 |  | 273 | 3.15 | benthos-plankton | demersal | freshwater |  |
| 25 | *Salmoniformes* | Salmoniformes |  | 128 |  |  |  |  |  | freshwater |  |
| 25 | *Coregonus lavaretus* | Salmoniformes |  | 106 |  | 273 | 3.15 | benthos-plankton | demersal | freshwater |  |
| 25 | *Salmoniformes* | Salmoniformes |  | 29 |  |  |  |  |  | freshwater |  |
| 25 | *Perca* | Perciformes |  |  |  | 200 | 3.66 | benthos | demersal | freshwater |  |
| 26 | *Esox lucius* | Esociformes | 771 | 1636 |  | 320 | 4.40 | plankton-benthos | demersal | freshwater |  |
| 26 | *Perca fluviatilis* | Perciformes | 1096 | 1571 |  | 200 | 3.66 | benthos | demersal | freshwater |  |
| 26 | *Abramis brama* | Cypriniformes | 512 | 571 |  | 157 | 3.15 | plankton-benthos | benthopelagic | freshwater |  |
| 27 | *Esox lucius* | Esociformes | 606 | 1189 |  | 320 | 4.40 | plankton-benthos | demersal | freshwater |  |
| 27 | *Lota lota* | Gadiformes | 598 |  |  | 432 | 4.03 | plankton-benthos | demersal | freshwater |  |
| 27 | *Perca fluviatilis* | Perciformes | 558 | 825 |  | 200 | 3.66 | benthos | demersal | freshwater |  |
| 28 | *Rutilus* | Cypriniformes |  | 147 |  | 194 | 2.87 | benthos-plankton | benthopelagic | freshwater |  |
| 28 | *Esox lucius* | Esociformes | 233 | 758 |  | 320 | 4.40 | plankton-benthos | demersal | freshwater |  |
| 28 | *Esox lucius* | Esociformes | 1577 | 581 |  | 320 | 4.40 | plankton-benthos | demersal | freshwater |  |
| 28 | *Perca fluviatilis* | Perciformes | 1218 | 385 |  | 200 | 3.66 | benthos | demersal | freshwater |  |
| 28 | *Esox lucius* | Perciformes | 457 | 385 |  | 320 | 4.40 | plankton-benthos | demersal | freshwater |  |
| 28 | *Esox lucius* | Esociformes | 307 | 385 |  | 320 | 4.40 | plankton-benthos | demersal | freshwater |  |
| 28 | *Perca fluviatilis* | Perciformes | 280 | 357 |  | 200 | 3.66 | benthos | demersal | freshwater |  |
| 28 | *Perca fluviatilis* | Perciformes | 171 | 357 |  | 200 | 3.66 | benthos | demersal | freshwater |  |
| 28 | *Perca fluviatilis* | Perciformes | 349 | 313 |  | 200 | 3.66 | benthos | demersal | freshwater |  |
| 28 | *Perca fluviatilis* | Perciformes | 404 | 313 |  | 200 | 3.66 | benthos | demersal | freshwater |  |
| 28 | *Salmo trutta* | Salmoniformes | 277 | 294 |  | 156 | 3.63 | plankton-benthos | demersal | freshwater |  |
| 28 | *Perca fluviatilis* | Perciformes | 168 | 263 |  | 200 | 3.66 | benthos | demersal | freshwater |  |
| 28 | *Esox lucius* | Esociformes | 168 | 263 |  | 320 | 4.40 | plankton-benthos | demersal | freshwater |  |
| 28 | *Salmo trutta* | Salmoniformes | 231 | 217 |  | 156 | 3.63 | plankton-benthos | demersal | freshwater |  |
| 28 | *Perca fluviatilis* | Perciformes | 241 | 208 |  | 200 | 3.66 | benthos | demersal | freshwater |  |
| 28 | *Salmo trutta* | Salmoniformes | 200 | 172 |  | 156 | 3.63 | plankton-benthos | demersal | freshwater |  |
| 28 | *Salmo trutta* | Salmoniformes | 126 | 128 |  | 156 | 3.63 | plankton-benthos | demersal | freshwater |  |
| 28 | *Perca fluviatilis* | Perciformes | 467 | 116 |  | 40 | 3.66 | benthos | demersal | freshwater |  |
| 28 | *Perca fluviatilis* | Perciformes | 240 | 114 |  | 200 | 3.66 | benthos | demersal | freshwater |  |
| 28 | *Salmo trutta* | Salmoniformes | 182 | 104 |  | 156 | 3.63 | plankton-benthos | demersal | freshwater |  |
| 28 | *Salmo trutta* | Salmoniformes | 222 |  |  | 156 | 3.63 | plankton-benthos | demersal | freshwater |  |
| 28 | *Perca fluviatilis* | Perciformes | 307 |  |  | 200 | 3.66 | benthos | demersal | freshwater |  |
| 28 | *Esox lucius* | Esociformes | 576 |  |  | 320 | 4.40 | plankton-benthos | demersal | freshwater |  |
| 29 | *Perca fluviatilis* | Perciformes | 1117 | 2652 |  | 200 | 3.66 | benthos | demersal | freshwater |  |
| 29 | *Perca fluviatilis* | Perciformes | 527 | 1792 |  | 200 | 3.66 | benthos | demersal | freshwater |  |
| 29 | *Perca fluviatilis* | Perciformes | 560 | 1353 |  | 200 | 3.66 | benthos | demersal | freshwater |  |
| 29 | *Perca fluviatilis* | Perciformes | 526 | 1572 |  | 200 | 3.66 | benthos | demersal | freshwater |  |
| 29 | *Esox lucius* | Esociformes | 548 | 2964 |  | 320 | 4.40 | plankton-benthos | demersal | freshwater |  |
| 29 | *Esox lucius* | Esociformes | 1078 | 1437 |  | 320 | 4.40 | plankton-benthos | demersal | freshwater |  |
| 29 | *Esox lucius* | Esociformes | 548 | 2060 |  | 320 | 4.40 | plankton-benthos | demersal | freshwater |  |
| 29 | *Esox lucius* | Esociformes | 548 | 4008 |  | 320 | 4.40 | plankton-benthos | demersal | freshwater |  |
| 30 | *Danio rerio* (*Brackydanio rerio*) | Cypriniformes |  | 51 |  | 0.25 | 3.21 | benthos | benthopelagic | freshwater | 26 |
| 31 | *Carassius auratus* | Cypriniformes |  | 80 |  | 4 | 2.86 | benthos | benthopelagic | freshwater | 12 |
| 31 | *Carassius auratus* | Cypriniformes |  | 71 |  | 4 | 2.86 | benthos | benthopelagic | freshwater | 12 |
| 31 | *Carassius auratus* | Cypriniformes |  | 55 |  | 4 | 2.86 | benthos | benthopelagic | freshwater | 26 |
| 31 | *Carassius auratus* | Cypriniformes |  | 50 |  | 4 | 2.86 | benthos | benthopelagic | freshwater | 20 |
| 31 | *Carassius auratus* | Cypriniformes |  | 33 |  | 4 | 2.86 | benthos | benthopelagic | freshwater | 20 |
| 31 | *Carassius auratus* | Cypriniformes |  | 33 |  | 4 | 2.86 | benthos | benthopelagic | freshwater | 28 |
| 31 | *Carassius auratus* | Cypriniformes |  | 19 |  | 4 | 2.86 | benthos | benthopelagic | freshwater | 28 |
| 32 | *Rutilus* | Cypriniformes |  | 192 |  | 194 | 2.87 | benthos-plankton | benthopelagic | freshwater | 14 |
| 32 | *Esox* | Esociformes |  | 152 |  | 320 | 4.40 | plankton-benthos | demersal | freshwater | 14 |
| 33 | *Perca fluviatilis* | Perciformes | 547 | 7337 | 0.0037 | 305 | 3.92 | benthos | demersal | freshwater | 14 |
| 33 | *Esox lucius* | Esociformes | 806 | 4271 | 0.0044 | 524 | 4.40 | plankton-benthos | demersal | freshwater | 14 |
| 33 | *Abramis brama* | Cypriniformes | 93 | 3030 | 0.0139 | 216 | 2.63 | plankton-benthos | benthopelagic | freshwater | 14 |
| 33 | *Perca fluviatilis* | Perciformes | 499 | 2774 | 0.0037 | 279 | 3.86 | benthos | demersal | freshwater | 14 |
| 33 | *Perca fluviatilis* | Perciformes | 97 | 2628 | 0.0084 | 15 | 3.09 | benthos | demersal | freshwater | 14 |
| 33 | *Esox lucius* | Esociformes | 723 | 2592 | 0.0004 | 743 | 4.40 | plankton-benthos | demersal | freshwater | 14 |
| 33 | *Perca fluviatilis* | Perciformes | 425 | 2409 | 0.0135 | 322 | 3.96 | benthos | demersal | freshwater | 14 |
| 33 | *Esox lucius* | Esociformes | 690 | 2044 | 0.0011 | 434 | 4.40 | plankton-benthos | demersal | freshwater | 14 |
| 33 | *Perca fluviatilis* | Perciformes | 66 | 2008 |  | 5 | 3.01 | benthos | demersal | freshwater | 14 |
| 33 | *Scardinius erytrophtalmus* | Cypriniformes | 154 | 1825 | 0.015 | 81 | 2.60 | benthos-plankton | benthopelagic | freshwater | 14 |
| 33 | *Abramis brama* | Cypriniformes | 235 | 1752 | 0.0037 | 200 | 2.89 | plankton-benthos | benthopelagic | freshwater | 14 |
| 33 | *Rutilus rutilus* | Cypriniformes | 166 | 1643 | 0.0102 | 24 | 2.47 | benthos-plankton | benthopelagic | freshwater | 14 |
| 33 | *Abramis brama* | Cypriniformes | 166 | 1606 | 0.0139 | 10 | 2.86 | plankton-benthos | benthopelagic | freshwater | 14 |
| 33 | *Rutilus rutilus* | Cypriniformes | 156 | 1533 | 0.0077 | 90 | 2.57 | benthos-plankton | benthopelagic | freshwater | 14 |
| 33 | *Carassius carassius* | Cypriniformes | 266 | 1424 | 0.0037 | 1008 | 3.00 | plankton-benthos | demersal | freshwater | 14 |
| 33 | *Perca fluviatilis* | Perciformes | 262 | 1351 | 0.0073 | 92 | 3.53 | benthos | demersal | freshwater | 14 |
| 33 | *Scardinius erytrophtalmus* | Cypriniformes | 156 | 1351 | 0.0099 | 71 | 2.38 | benthos-plankton | benthopelagic | freshwater | 14 |
| 33 | *Gymnocephalus cernuus* (*Acerina cernua*) | Perciformes | 115 | 1351 | 0.0073 | 8.4 | 3.00 | plankton-benthos | demersal | freshwater | 14 |
| 33 | *Gymnocephalus cernuus* (*Acerina cernua*) | Perciformes | 132 | 1278 | 0.0004 | 7 | 3.00 | plankton-benthos | demersal | freshwater | 14 |
| 33 | *Abramis brama* | Cypriniformes | 193 | 1278 | 0.0004 | 141 | 2.93 | plankton-benthos | benthopelagic | freshwater | 14 |
| 33 | *Abramis brama* | Cypriniformes | 114 | 1205 | 0.0024 | 12 | 2.56 | plankton-benthos | benthopelagic | freshwater | 14 |
| 33 | *Rutilus rutilus* | Cypriniformes | 140 | 1205 | 0.0146 | 24 | 2.61 | benthos-plankton | benthopelagic | freshwater | 14 |
| 33 | *Rutilus rutilus* | Cypriniformes | 158 | 1168 | 0.0117 | 25 | 2.65 | benthos-plankton | benthopelagic | freshwater | 14 |
| 33 | *Rutilus rutilus* | Cypriniformes | 152 | 1132 | 0.0234 | 6 | 2.74 | benthos-plankton | benthopelagic | freshwater | 14 |
| 33 | *Perca fluviatilis* | Perciformes | 258 | 1059 | 0.0102 | 73 | 3.42 | benthos | demersal | freshwater | 14 |
| 33 | *Gymnocephalus cernuus* (*Acerina cernua*) | Perciformes | 150 | 1022 | 0.0084 | 9 | 3.00 | plankton-benthos | demersal | freshwater | 14 |
| 33 | *Perca fluviatilis* | Perciformes | 193 | 986 | 0.0128 | 80 | 3.54 | benthos | demersal | freshwater | 14 |
| 33 | *Rutilus rutilus* | Cypriniformes | 112 | 986 | 0.008 | 8 | 2.55 | benthos-plankton | benthopelagic | freshwater | 14 |
| 33 | *Rutilus rutilus* | Cypriniformes | 135 | 949 | 0.0037 | 86 | 2.46 | benthos-plankton | benthopelagic | freshwater | 14 |
| 33 | *Perca fluviatilis* | Perciformes | 56 | 876 | 0.0121 | 6 | 3.01 | benthos | demersal | freshwater | 14 |
| 33 | *Rutilus rutilus* | Cypriniformes | 224 | 840 | 0.0031 | 85 | 2.57 | benthos-plankton | benthopelagic | freshwater | 14 |
| 33 | *Abramis brama* | Cypriniformes | 228 | 803 | 0.0004 | 6 | 2.98 | plankton-benthos | benthopelagic | freshwater | 14 |
| 33 | *Alburnus lucidus* | Cypriniformes | 164 | 767 | 0.0058 | 13 | 2.95 | plankton-benthos | benthopelagic | freshwater | 14 |
| 33 | *Perca fluviatilis* | Perciformes | 227 | 767 | 0.0084 | 18 | 3.06 | benthos | demersal | freshwater | 14 |
| 33 | *Perca fluviatilis* | Perciformes | 169 | 694 | 0.0124 | 6 | 3.01 | benthos | demersal | freshwater | 14 |
| 33 | *Perca fluviatilis* | Perciformes | 87 | 657 | 0.0036 | 17 | 3.07 | benthos | demersal | freshwater | 14 |
| 33 | *Rutilus rutilus* | Cypriniformes | 107 | 548 | 0.0139 | 7 | 2.81 | benthos-plankton | benthopelagic | freshwater | 14 |
| 34 | *Salmo trutta* | Salmoniformes |  | 564 | 0.0012 | 416 | 3.63 | plankton-benthos | demersal | freshwater | 4.4 |
| 34 | *Salmo trutta* | Salmoniformes |  | 481 | 0.0014 | 323 | 3.63 | plankton-benthos | demersal | freshwater | 4.4 |
| 34 | *Salmo trutta* | Salmoniformes |  | 363 | 0.0019 | 300 | 3.63 | plankton-benthos | demersal | freshwater | 8.6 |
| 34 | *Salmo trutta* | Salmoniformes |  | 345 | 0.0021 | 472 | 3.63 | plankton-benthos | demersal | freshwater | 8.6 |
| 34 | *Salmo trutta* | Salmoniformes |  | 338 | 0.0021 | 49 | 3.63 | plankton-benthos | demersal | freshwater | 4.4 |
| 34 | *Salmo trutta* | Salmoniformes |  | 221 | 0.0031 | 496 | 3.63 | plankton-benthos | demersal | freshwater | 11.7 |
| 34 | *Salmo trutta* | Salmoniformes |  | 214 | 0.0032 | 53 | 3.63 | plankton-benthos | demersal | freshwater | 8.6 |
| 34 | *Salmo trutta* | Salmoniformes |  | 208 | 0.0033 | 203 | 3.63 | plankton-benthos | demersal | freshwater | 11.7 |
| 34 | *Salmo trutta* | Salmoniformes |  | 167 | 0.0042 | 47 | 3.63 | plankton-benthos | demersal | freshwater | 11.7 |
| 34 | *Salmo trutta* | Salmoniformes |  | 162 | 0.0043 | 454 | 3.63 | plankton-benthos | demersal | freshwater | 15.3 |
| 34 | *Salmo trutta* | Salmoniformes |  | 140 | 0.005 | 119 | 3.63 | plankton-benthos | demersal | freshwater | 15.6 |
| 34 | *Salmo trutta* | Salmoniformes |  | 140 | 0.005 | 220 | 3.63 | plankton-benthos | demersal | freshwater | 15.6 |
| 34 | *Salmo trutta* | Salmoniformes |  | 104 | 0.0067 | 23 | 3.63 | plankton-benthos | demersal | freshwater | 15.5 |
| 35 | *Esox lucius* | Esociformes |  | 475 |  | 500 | 4.40 | plankton-benthos | demersal | freshwater | 9 |
| 35 | *Lutjanus argentimaculatus* | Perciformes |  | 34 | 0.022 | 14 | 3.85 | plankton-benthos | demersal | marine | 25 |

References

1. Baptist JP, Price TJ (1962) Accumulation and retention of cesium137 by marine fishes. In: Fish and Wildlife Service, Superintendent of Documents, US Govt.

2. Baudin J (2000) Dietary uptake, retention and tissue distribution of ^54^Mn, ^60^Co and ^137^Cs in the rainbow trout (*Oncorhynchus mikiss* Walbaum). Wat Res 34:2869-2878.

3. Brittain JE, Gjerseth JE (2010) Long-term trends and variation in ^137^Cs activity concentrations in brown trout (*Salmo trutta*) from Øvre Heimdalsvatn, a Norwegian subalpine lake. Hydrobiologia 642:107-113.

4. Broberg A, Andersson E (1991) Distribution and circulation of Cs-137 in lake ecosystems. In: Moberg L, editor. The Chernobyl Fallout in Sweden, Swedish Radiation Protection Institute, Stockholm, Sweden. pp. 151-175.

5. Carlsson S (1978) A model for the turnover of ^137^Cs and potassium in pike (*Esox lucius*). Health Phys 35:549-554.

6. Elliott JM, Elliott JA, Hilton J (1993) Sources of variation in post-Chernobyl radiocaesium in brown trout, *Salmo trutta* L., and Arctic charr, *Salvelinus alpinus* (L.), from six Cumbrian lakes (nothwest England). Annls Limnol 29: 79-98.

7. Elliott JM, Hilton J, Rigg E, Tullett PA, Swift DJ, et al. (1992) Sources of variation in post-Chernobyl radiocaesium in fish from two Cumbrian lakes (north-west England). J Appl Ecol 29:108-119.

8 Forseth T, Ugedal O, Jonsson B, Langeland A, Njåstad O (1991) Radiocaesium turnover in Arctic charr (*Salvelinus alpinus*) and brown trout (*Salmo trutta*) in a Norwegian lake. J Appl Ecol 28:1053-1067.

9. Gallegos AF, Whicker FW (1971) Radionuclides in ecosystems. Proceedings of the Third National Symposium on Radioecology, Oak Ridge, Tennesee. pp 361-371.

10. Hammar, J., Notter, M., Neumann G (1991) Northern reservoirs as sinks for Chernobyl cesium: sustained accumulation via introduced *Mysis relicta* in Arctic charr and Brown trout. In: Moberg L, editor. The Chernobyl Fallout in Sweden, Swedish Radiation Protection Institute, Stockholm, Sweden. pp 183-205.

11. Hanson WC (1967) Radioecological Concentration Processes, Pergamon Press, Oxford. pp 183-191.

12. Håkanson L, Andersson T (1992) Remedial measures against radioactive caesium in Swedish lake fish after Chernobyl. Aquat Sci 54:141-164.

13. Kevern NR (1966) Feeding rate of carp estimated by a radioisotropic method. Tran Ame Fish Soc 95:363-371.

14. Kolehmainen SE (1974) The balance of ^137^Cs, stable cesium and potassium of bluegill (*Lepomis macrochirus* Raf.) and other fish in White Oak Lake. Health Phys 23:301-315.

15. Koulikov AO, Ryabov IN (1992) Specific cesium activity in freshwater fish and the size effect. Sci Total Env 112:125-142.

16. Kritidis P, Florou H (1995) Environmental study of radioactive caesium in Greek lake fish after the Chernobyl accident. J Environ Radioactivity 28:285-293.

17. Kryshev I (1995) Radioactive contamination of aquatic ecosystems following the Chernobyl accident. J Environ Radioactivity 27:207-219.

18. Malek MA, Nakahara M, Nakamura R (2004) Uptake, retention and organ/tissue distribution of ^137^Cs by Japanese catfish (*Silurus asotus* Linnaeus). J Environ Radioactivity 77:191-204.

19. Morgan I, Tytler P, Bell M (1993) The accumulation of 137-caesium from fresh water by alevins and fry of Atlantic salmon and brown trout. J Fish Biol 43:877-888.

20. Peles JD, Bryan a L, Garten CT, Ribble DO, Smith MH (2000) Ecological half-life of ^137^Cs in fish from a stream contaminated by nuclear reactor effluents.  Sci Total Environ 263:255-262.

21. Perston A, Jefferies DF, Dutton JWR (1967) The concentrations of caesium-137 and strontium-90 in the flesh of brown trout taken from rivers and lakes in the British Isles between 1961 and 1966: the variables determining the concentrations and their use in radiological assessments. Wat Res 1:475-496.

22. Peters EL, Newman MC (1999) ^137^Cs elimination by chronically-contaminated largemouth bass (*Micropterus salmoides*). Health Phys 76:260-268.

23. Peters EL, Schultz IR, Newman MC (1999) Rubidium and cesium kinetics and tissue distributions in channel catfish (*Ictalurus punctatus*). Ecotoxicology 8:287-300.

24. Pinder JE, Hinton TG, Whicker FW, Smith JT (2009) Cesium accumulation by fish following acute input to lakes: a comparison of experimental and Chernobyl-impacted systems. J Environ Radioactivity 100:456-467.

25. Pröhl G, Ehlken S, Fiedler I, Kirchner G, Klemt E, et al. (2006) Ecological half-lives of ^90^Sr and ^137^Cs in terrestrial and aquatic ecosystems. J Environ Radioactivity 91:41-72.

26. Rask M, Saxén R, Ruuhijärvi J, Arvola L, Järvinen M, et al. (2012) Short- and long-term patterns of ^137^Cs in fish and other aquatic organisms of small forest lakes in southern Finland since the Chernobyl accident. J Environ Radioactivity 103:41-47.

27. Ryabov IN (2002) Long-term observation of radioactivity contamination in fish around Chernobyl. Recent research activities about the Chernobyl NPP accident in Belarus, Ukraine and Russia. KURRI-KR-79:112-122.

28. Saxén R, Ilus E (2008) Transfer and behaviour of ^137^Cs in two Finnish lakes and their catchments. Sci Total Env 394:349-360.

29. Smith JT, Kudelsky AV, Ryabov IN, Daire SEA, Boyer L, et al. (2002) Uptake and elimination of radiocaesium in fish and the “size effect”. J Environ Radioactivity 62:145-164.

30. Srivastava A, Denschlag HO, Kelber O, Urich K (1990) Accumulation and discharge behavior of Cs-137 by zebra fish (*Brachydanio rerio*) in different aquatic environment. J Radioanal Nucl Chem Artic 138:165-170.

31. Srivastava A, Reddy SJ, Kelber O, Urich K, Denschlag HO (1994) Uptake and release kinetics of ^134^Cs by goldfish (*Carassius auratus*) and ^137^Cs by zebra fish (*Brachydanio rerio*) in controlled aquatic environment. J Radioanal Nucl Chem Artic 182:63-69.

32. Sundblad B, Bergstom U, Evans S (1991) Long term transfer of fallout nuclides from the terrestrial to the aquatic environment. Evaluation of ecological models. In: Moberg L, editor. The Chernobyl Fallout in Sweden, Swedish Radiation Protection Institute, Stockholm, Sweden. pp 207–238.

33. Sundbom M, Meili M, Andersson E, Östlund M, Broberg A (2003) Long-term dynamics of Chernobyl ^137^Cs in freshwater fish: quantifying the effect of body size and trophic level. J Appl Ecol 40:228-240.

34. Ugedal O, Jonsson B, Njastad O, Naeumann R (1992) Effects of temperature and body size on radiocaesium retention in brown trout, *Salmo trutta*. Freshw Biol 28:165-171.

35. Zhao X, Wang W, Yu K, Lam P (2001) Biomagnification of radiocesium in a marine piscivorous fish. Mar Ecol Prog Ser 222:227-237.
